# An integrated microfluidic system for automatic and self-validated analysis of cervical extracellular vesicle markers PD-L1 and ERBB3

**DOI:** 10.1007/s44211-026-00871-8

**Published:** 2026-03-16

**Authors:** Yunxing Lu, Han Qin, Wenjing Zhang, Qiang Shi, Jianan Hui, Zhenhua Wu, Yiman Song, Xiaoyue Yang

**Affiliations:** 1 School of Science and Technology, Shanghai Open University, Shanghai, 200433 China; 2 The International Peace Maternity and Child Health Hospital, School of Medicine, Shanghai Jiao Tong University, Shanghai, 200030 China; 3 Department of Obstetrics and Gynecology, The Second Affiliated Hospital of Soochow University, Suzhou, 215000 China; 4 State Key Laboratory of Transducer Technology, Shanghai Institute of Microsystem and Information Technology, Chinese Academy of Sciences, Shanghai, 200050 China; 5 Shanghai Frontier Innovation Research Institute, Shanghai, China; 6 School of Stomatology, Dalian Medical University, Dalian, 116044 China; 7 Shanghai Key Laboratory of Embryo Original Diseases, Shanghai, 200030 China

**Keywords:** Extracellular vesicles, Integrated microfluidic system, Deep learning, Immune evasion, Proliferative signaling

## Abstract

The early and precise diagnosis of gynecological malignancies, such as cervical cancer, is critical for improving patient treatments. Extracellular vesicles (EVs), such as exosomes, which carry molecular signals from their parental cells, offer a promising method for non-invasive liquid biopsy, however, conventional detection methods are often complex, high in reagent consumption, and susceptible to environmental fluctuations. To address this, we present an integrated, self-validated microfluidic system for the rapid, on-chip isolation and multiplexed identification of the gynecological EV markers PD-L1 and ERBB3. The chip achieved simultaneous on-chip processing of test and positive samples for parallel analysis within 1 h, enabling synchronous detection under the same conditions and thereby significantly enhancing the reliability of the assay. Additionally, a deep learning YOLOv8-based self-validated detection strategy facilitates automated and precise fluorescence identification. Validation with four cell lines (SiHa, C33A, HeLa, and H8) revealed remarkable EV protein signatures, achieving a limit of detection (LOD) of 15.56 particles/μL. This platform provides an integrated tool for sensitive and precise EV marker analysis, holding prospective potential for the early screening and personalized therapy guidance of gynecological tumor detection.

**Graphical abstract:**

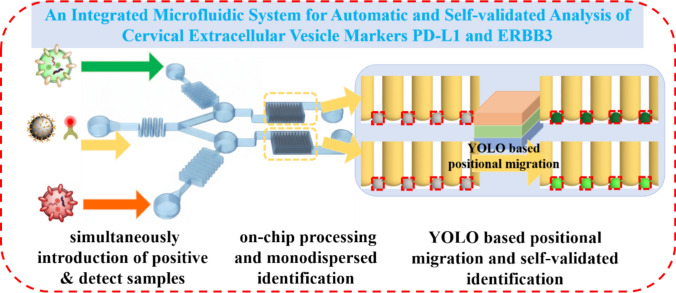

Integrated analytical system for one-stop and self-validated exosome complex formation and multiplex tumor fingerprint analysis by deep learning. Exosome samples were immuno-isolated and labeled with probes, followed by monodispersed among the particular arrays for deep learning model YOLOv8-based positional migration and identification automatically. Four kinds of samples were measured and remarkable differences were acquired, the two tumor progressions: immune evasion and proliferative signaling were revealed. The integrated system is pospective for sensitive, easy-handing and automatic exosome markers analysis in POCT field.

**Supplementary Information:**

The online version contains supplementary material available at 10.1007/s44211-026-00871-8.

## Introduction

Gynecological malignancies, with cervical cancer being one of the most prevalent worldwide, pose a significant threat to women’s health [1, 2]. The etiology of cervical cancer is strongly associated with persistent infection by high-risk human papillomavirus (HPV). The development and progression of these tumors involve complex molecular mechanisms, including the aberrant activation of signaling pathways and profound immune evasion [3–5].

Extracellular vesicles (EVs) are cell-derived membrane-bound nanovesicles typically ranging from 30 to 200 nm in diameter. Among these, exosomes, which originate from the endosomal pathway, play a pivotal role in intercellular communication [6–9]. By transferring a diverse cargo of bioactive molecules—including proteins, lipids, and nucleic acids—they modulate a wide array of physiological and pathological processes. In the field of oncology, tumor-derived EVs (often enriched for exosomes) are particularly significant as they carry molecular markers reflective of their parental cells, contributing to tumor progression, immune suppression, and the establishment of pre-metastatic niches [10–12]. The inherent stability of EVs in biofluids and their relative abundance make them exceptionally attractive biomarkers for liquid biopsy [11, 13].

Within the rich EV proteome, specific markers hold high clinical relevance. Programmed Death-Ligand 1 (PD-L1), an immune checkpoint protein, is instrumental in tumor-mediated immunosuppression. When expressed on EVs, PD-L1 can bind to the PD-1 receptor on activated T-cells, inducing T-cell anergy or apoptosis and allowing the tumor to evade immune surveillance [14–16]. Consequently, the level of EV-associated PD-L1 can serve as a systemic indicator of a tumor’s immunosuppressive activity. Concurrently, receptor tyrosine-protein kinase ERBB3 (also known as HER3), a member of the epidermal growth factor receptor (EGFR) family, is frequently overexpressed in various cancers and is strongly associated with proliferation, therapeutic resistance, and poor prognosis. ERBB3 and PD-L1 represent two critical axes of malignancy: intrinsic proliferative signaling and extrinsic immune evasion, respectively. Therefore, the multiplexed quantification of these two markers on EVs can provide a more holistic and functionally relevant snapshot of a tumor’s biological state, offering superior diagnostic and prognostic power [7, 17].

Despite the promise, the clinical translation of EV-based diagnostics is facing persistent challenges in isolation and analysis. Gold-standard techniques such as ultracentrifugation are not only costly and labor-intensive but also yield products with potential contaminants, including protein aggregates and other vesicles of similar size. Subsequent analytical methods, including ELISA and western blotting, typically demand large sample volumes and involve multiple, protracted steps, rendering them unsuitable for rapid, point-of-care diagnostics [18–21]. Microfluidic technology has emerged as a powerful alternative, leveraging scale effects to enhance sensitivity while reducing reagent consumption and reaction times [22–28]. However, existing microfluidic strategies face their own shortcomings, such as complex fabrication protocols, susceptibility to channel clogging, and, critically, measurement variability arising from batch-to-batch inconsistencies [20, 29–32].

In recent years, the integration of deep learning has introduced a new dimension to microfluidic analysis, demonstrating immense potential in processing and analyzing original data [33–36]. Advanced deep learning models, particularly convolutional neural networks (CNN) like the YOLO (You Only Look Once) architecture, are exceptionally adept at object detection and image segmentation. By training models on public or custom datasets of raw images, they can learn to automatically identify and classify interested objects with high accuracy and efficiency, significantly reducing the potential for human bias and error inherent in manual quantification. This automated approach is crucial for achieving the sensitive, accurate, and unbiased results required for clinical applications [37–39]. The integration of deep learning with microfluidics has shown promise in cell counting, classification and detection. However, the application of advanced object detection models, for the automated, high-throughput, together with unbiased quantification of fluorescence intensity on microbeads remains underexplored [39–43]. In our recent work [44], we explored a deep-learning-enabled microfluidic workflow for exosomal analysis. While effective, that system relied on sequential batch processing, which can be susceptible to environmental fluctuations and intra-chip variations.

In this research, we designed and validated an integrated, self-validated microfluidic platform for the rapid, multiplexed analysis of EV-associated PD-L1 and ERBB3. The core innovation of our system is its on-chip self-validated design. Unlike conventional microfluidic devices that rely on batch-to-batch calibration, our platform employs a novel triple-inlet, dual-channel architecture designed specifically for real-time validation. By introducing the test sample and a positive reference simultaneously alongside a shared reagent stream, this symmetric configuration ensures that both samples undergo identical processing conditions. This integration effectively eliminates intra-run variations and systematic errors often encountered in sequential analysis, thereby significantly enhancing the reliability of the assay. Furthermore, a YOLOv8-based deep learning algorithm is utilized for automated, unbiased quantification of fluorescent signals from individual microbeads with high precision. Besides, a ‘positional migration’ strategy is executed for objective signal quantification, eliminating human bias, which extends the algorithm’s capability from simple recognition to precise quantitative analysis. We validated the platform by profiling EVs from cervical cancer cell lines with different HPV statuses and pathologies (SiHa, C33A, HeLa) against a normal cervical epithelial cell line (H8), demonstrating its capability for rapid and differential tumor profiling.

## Materials and methods

### Reagents and materials

Polydimethylsiloxane (PDMS, Sylgard 184) was obtained from Dow Corning Co. (NY, USA). The chemical reagents 1-ethyl-3-[3-dimethylaminopropyl] carbodiimide (EDC), Sulfo-N-hydroxysulfosuccinimide (NHS), and trichloro(1H,1H,2H,2H-perfluorooctyl) silane (PFOTS) were acquired from Sigma-Aldrich (St. Louis, MO, USA). Surfactant (Tween-20) and Phosphate-buffered saline (PBS) were products of Sangon Biotech. Bovine serum albumin (BSA) was purchased from Equitech-Bio-Inc. Carboxylic-functionalized magnetic beads (15 µm diameter, consisting of a polystyrene core with an iron oxide-infused outer layer) were sourced from BaseLine ChromTech Research Center (Tianjin, China). Anti-CD63 antibody (for capture), Fluor488-labeled anti-PD-L1 antibody, and anti-ERBB3 antibody were purchased from Abcam (UK). The cell lines SiHa, C33A, HeLa, and H8 were procured from the Cell Bank of the Chinese Academy of Sciences (Shanghai, China). Fetal bovine serum (FBS) and cell culture media (RPMI 1640, MEM, DMEM) were from Gibco Thermo Fisher Scientific (Waltham, MA, USA).

For Western blot analysis of EVs, Western lysis buffer and PMSF were purchased from Beyotime Biotechnology (Shanghai, China). The Pierce™ BCA Protein Assay Kit and PageRuler™ Prestained Protein Ladder were obtained from Thermo Fisher Scientific (Waltham, MA, USA). Primary antibodies against PD-L1 (ab228415) were sourced from Abcam (Cambridge, UK). HRP-labeled goat anti-rabbit (A0208) and goat anti-mouse (A0216) IgG (H+L) secondary antibodies were acquired from Beyotime Biotechnology. The LumiBest ECL substrate (SB-WB011) was purchased from Share-bio (Shanghai, China).

### Microfluidic system design and fabrication

The PDMS microchip was fabricated using standard soft lithography protocols. The chip layout was designed in AutoCAD and transferred to a chromium photomask. A key feature of the design is a triple-inlet structure, where two side inlets introduce the test sample and positive reference, and the central inlet introduces the reagent mixture. The fluids converge and are split into two independent serpentine mixing channels. These channels are designed to increase hydrodynamic resistance, which stabilizes the flow profile, ensures balanced and thorough mixing of the converging streams, and prevents pressure-induced backflow during sample loading. They are connected to two downstream circular incubation chambers. The analysis region consists of two micropillar arrays engineered to generate oblique liquid flow for monodispersed trapping of test beads and positive reference beads, respectively, thereby facilitating subsequent image acquisition and data analysis. The microchannels were designed with a uniform depth of 20 µm and a width of 100 µm. The micropillars in the detection zone have a diameter of 50 µm with an inter-pillar spacing of 14 µm, optimized to physically trap the 15 µm magnetic beads while allowing fluid flow.

The pattern was used to create a master mold on a silicon wafer via photolithography and deep reactive ion etching (deepRIE). To create an anti-adhesive surface, the silicon master was silanized by vapor-phase deposition of PFOTS for 12 h under vacuum. A PDMS prepolymer and curing agent mixture (10:1 w/w ratio) was poured over the master, degassed, and cured at 95 °C for 2 h. After curing, inlet and outlet ports were punched. The structured PDMS layer and a coverglass were then treated with air plasma before being irreversibly bonded to form the final device.

### Preparation of anti-CD63 immuno-magnetic beads

Magnetic beads were functionalized with anti-CD63 capture antibodies to facilitate EV isolation. 20 µL of beads was first suspended in 80 µL of PBS and washed. For carboxyl group activation, the beads were incubated in a solution containing 10 mg/mL Sulfo-NHS and 10 mg/mL EDC for 2 h at 25 °C under acidic conditions. After that, the beads were pelleted and re-suspended in a 100 µL PBS solution containing 10 µg/mL of the anti-CD63 antibody and incubated for 2 h at 37 °C to facilitate covalent bond formation. A final wash removed unbound antibodies, and the conjugated beads were resuspended in 100 µL of a blocking buffer (PBS-TB: 0.1% BSA and 0.02% Tween-20 in PBS) and stored at 4 °C until use. The final working concentration of beads used for on-chip assays was about 5 × 10^4^ beads/mL.

### Cell culture and supernatant preparation

The cell lines SiHa, C33A, HeLa, and H8 were confirmed to be mycoplasma-free and maintained in a Forma direct-heat CO₂ incubator (Thermo Fisher Scientific, Waltham, MA, USA) at 37 °C with 5% CO_2_. SiHa and C33A cells were cultured in MEM, HeLa cells in DMEM, and H8 cells in RPMI 1640. All culture media were supplemented with 10% (v/v) FBS and 1% penicillin–streptomycin. Cells were passaged every 2–3 days upon reaching 80–90% confluence. For EVs collection, upon reaching 80% confluence, cultured cells were washed twice with PBS and switched to a serum-free medium to eliminate interference from bovine EVs present in FBS. The conditioned medium (~ 10–20 mL per flask) was collected after 48 h of starvation culture. The collected medium was immediately processed or stored at − 80 °C. To prepare the supernatant for on-chip analysis, the conditioned medium underwent sequential centrifugation at 4 °C: first at 2000×  g for 30 min to pellet cells, followed by 10,000×  g for 30 min to remove larger debris and apoptotic bodies. The resulting supernatant was carefully collected and used for subsequent experiments.

### Isolation of model EV samples

For validation and characterization experiments, EVs were isolated from cell culture supernatant via ultracentrifugation. Once the cultured cells reached 80% confluence, the medium was replaced with serum-free medium. The conditioned medium (approximately 50 mL) was harvested after starvation culture for 48 h. The isolation process consisted of a series of centrifugation steps performed at 4 °C: an initial spin at 2000×  g for 30 min to pellet cells, followed by centrifugation at 10,000×  g for 30 min to eliminate larger debris. The resulting supernatant was then ultracentrifuged at 100,000 ×  g for 70 min and repeated once to improve purity. Finally, the EV pellet was resuspended in 100 µL of sterile PBS.

### On-chip immunoassay procedure

The total immunoassay was performed on-chip within one hour. First, the channels were primed with PBS-TB to minimize non-specific surface adsorption. To ensure standardized sample input for the microfluidic assay, the size distribution and concentration of EVs isolated from the four cell lines were characterized using a NanoSight NS300 (Malvern Panalytical) and ZetaView (PMX120). Prior to analysis, the concentration of all cell supernatant-derived EV samples was quantified by NTA, and samples were normalized to a concentration of 1 × 10^9^ particles/mL to ensure uniform EV input for all on-chip assays and minimize input variation.

The test sample and positive reference sample (SiHa supernatant, selected for its consistently high marker expression) were injected using high-precision syringe pumps (Harvard Pump 11 Pico Plus Elite) through the two side inlets at 1 µL/min, while a reagent cocktail containing anti-CD63 beads (about 5 × 10^4^ beads/mL) and fluorescent probes (final concentration 1 µg/mL) was injected through the central inlet. A co-incubation strategy was employed, where beads, EVs, and probes interact simultaneously and enhanced reaction kinetics provided by the microfluidic environment. Unlike off-chip sequential methods that require multiple washing or centrifugation steps, the high surface-to-volume ratio of the micro-chambers facilitates rapid mass transfer and efficient immune complex formation within a condensed timeframe. The solutions were infused continuously at 1 µL/min for 10 min, resulting in a total injected volume of 10 µL per inlet and incubated in-situ for 30 min. Following this, a washing buffer (PBS-TB) was introduced to flush out unbound reagents to mitigate potential non-specific adsorption of EVs or reagents to the PDMS channel walls. After the wash, the magnet was removed, and the immuno-complexes were directed downstreamed into the micropillar array, where they were monodispersed in respective detection zones for self-validated imaging and identification.

It is worthy noting that the symmetric dual-channel design serves as an intrinsic correction mechanism; any residual non-specific loss affects both the test and positive reference channels equally and is effectively normalized in the final ratiometric analysis.

To verify the specificity of the assay, control experiments were performed (see Fig. S1), including incubating non-functionalized beads with EV-positive samples (bead specificity control) and functionalized beads with EV-free medium (probe specificity control). Both controls showed negligible fluorescence, confirming minimal non-specific adsorption.

The SiHa supernatant was chosen as the positive reference because our preliminary screening confirmed it consistently expresses high levels of both target markers (PD-L1 and ERBB3), making it an ideal on-chip positive control for normalizing the entire assay and validating each performance of experimental runs.

### Western blot analysis

To validate the on-chip profiling results, Western blot analysis was performed on the lysates of EVs (SiHa, C33A, HeLa, and H8). Total protein was extracted using Western lysis buffer supplemented with PMSF, and protein concentration was determined via BCA assay. Equal amounts of protein were separated by SDS-PAGE using a vertical electrophoresis cell and transferred to membranes. The membranes were incubated overnight with primary antibodies against PD-L1 (1:1000), followed by HRP-conjugated secondary antibodies. Protein bands were visualized using an ECL detection system and quantified to corroborate the EV marker profiles obtained from the microfluidic chip.

### Image acquisition and automated data analysis

Bright-field and fluorescence micrographs of each sample were acquired through an inverted fluorescence microscope using a 20×  objective lens. The exposure time was consistently set at 1.8 s, and the black balance value was 1800 for all parallel experiment sets. Quantitative analysis was performed using a custom-trained YOLOv8 deep learning model. The YOLOv8 architecture consists of three key components: a Backbone for efficient feature extraction, a Neck that fuses object features from different scales to enrich contextual information, and a Head that performs the final detection. For model training, a dataset of approximately 10,000 microbead micrographs was manually annotated using LabelImg to create bounding boxes. The dataset was partitioned into training, validation, and test sets at a 7:2:1 ratio, and the model was trained using an Adam optimizer with an initial learning rate of 0.001. Data augmentation techniques (rotation, flipping) were applied to enhance robustness. The model was trained for 300 epochs using an Adam optimizer. Performance validation in Fig. S2 ensuring reliable bead recognition.

The model implements a “positional migration” strategy for unbiased quantification. First, the algorithm processes the bright-field image to accurately identify the location and boundaries of every microbead. Second, these coordinates are programmatically transferred to the fluorescence image, and the mean grayscale intensity is calculated exclusively within these pre-defined boundaries. The software then automatically calculates the mean fluorescence intensity for both zones and outputs the final Relative Fluorescence Ratio (RFR) by dividing the Value of Mean Detection Fluorescence (V_MDF_) by the Value of Mean Positive Fluorescence (V_MPF_) as follows:

$${\text{RFR }} = \frac{{{\mathrm{VMDF}}}}{{{\mathrm{VMPF}}}} * 100\%$$

The final fluorescence value for each channel (test or positive reference) was calculated as the average of the mean grayscale intensities from all individual microbeads identified by the YOLOv8 model within that detection zone.

For SEM imaging, after the on-chip reaction was completed, inlets were sealed, and the PDMS was punctured over the incubation chamber. Beads were retrieved by back-flushing with PBS, deposited on a silicon wafer, dried, and sputter-coated for imaging.

## Results and discussion

### Principle of the integrated self-validated microfluidic system

**Fig. 1:**
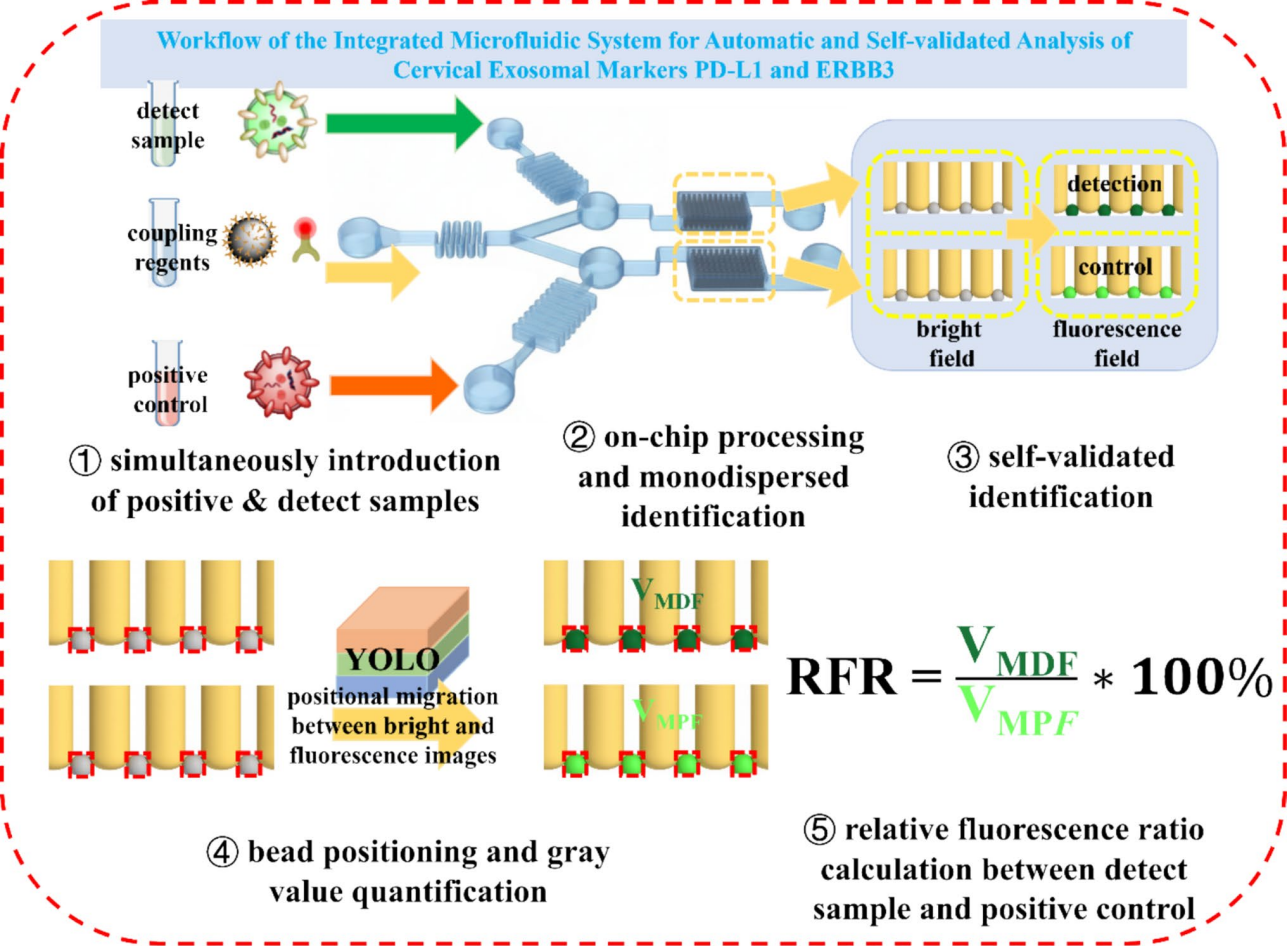
Schematic of the overall working principle of the chip. It integrates on-chip sample processing and detection, and ensures the validity of the detection results through simultaneous analysis with a positive reference. A YOLO-based microbead recognition framework combined with a “position migration” algorithm achieves grayscale analysis by identifying bead positions in the bright-field and analyzing the corresponding fluorescence field. Grayscale information is ultimately presented as a Relative Fluorescence Ratio (RFR) relative to the positive reference

The operational workflow is designed for an integrated “sample-in, answer-out” analysis, as illustrated in the schematic in Fig. 1. The core of the system is a microfluidic chip engineered for simultaneous, parallel processing to ensure result validity. Utilizing a triple-inlet, dual-channel architecture, the device simultaneously introduces a test sample and a positive reference sample (this study used SiHa-derived EVs as the positive reference) through its side inlets. Concurrently, the central inlet injects a reagent cocktail containing anti-CD63 magnetic beads for EV capture and fluorescently labeled probes targeting the biomarkers PD-L1 and ERBB3. This process involves the on-chip mixing of samples with the reagent cocktail, followed by in-situ incubation where EV-bead-label immuno-complexes are formed. The “answer-out” component is facilitated by a YOLOv8-based deep learning algorithm, which employs a “positional migration” strategy to identify microbead locations in the bright-field image and quantify their corresponding grayscale intensity in the fluorescence image. The final output, a normalized relative fluorescence ratio (RFR), provides a reliable measure of biomarker expression. This self-validated design ensures that the test sample and the positive reference undergo identical processing in parallel within the same chip, at the same time, and with the same reagent batch, effectively eliminating systematic errors caused by operational variability, reagent degradation, or environmental fluctuations.

The schematic is a 2D top-down view, illustrating how microbeads are hydrodynamically trapped by the micropillar array. The immobilization of microbeads within the detection zone is governed by a hydrodynamic trapping mechanism rather than gravitational sedimentation. The injected beads were leaded by lateral and longitudinal velocity gradients generated from micropillar array. The occupancy of a trap by a single microbead would increases the local hydraulic resistance along the longitudinal path, while the lateral flow component remains unobstructed. This resistance disparity actively diverts subsequent beads laterally toward adjacent empty traps, thereby facilitating a self-regulated, sequential loading process that ensures high-efficiency monodispersion across the array.

**Fig. 2:**
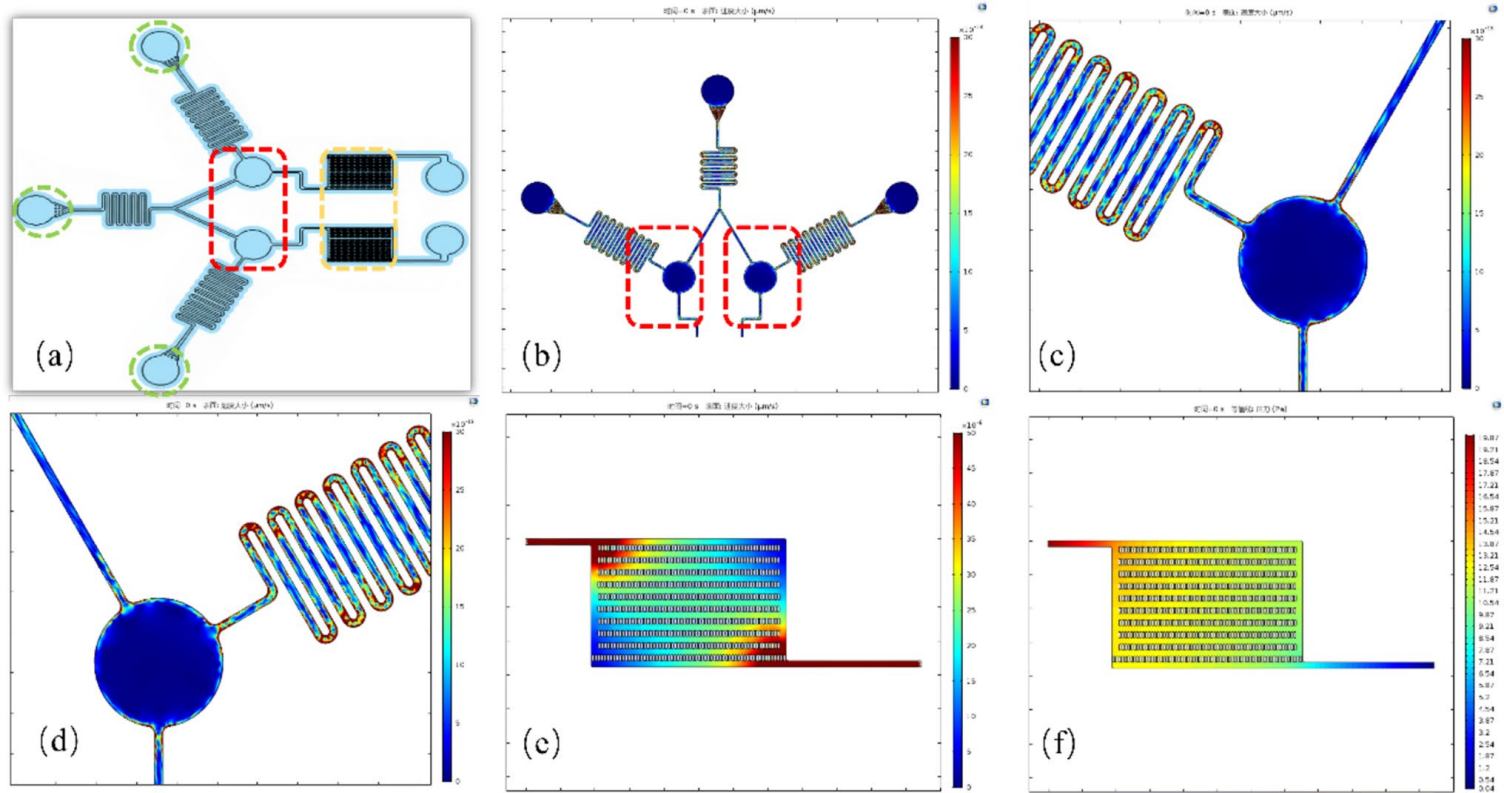
Simulation results of the chip’s functional structures. **a** Chip structure diagram, where green circles indicate the 3 inlets, the red box shows the on-chip incubation chambers, and the yellow box indicates the monodisperse detection region. **b** COMSOL simulation showing the velocity field distribution in the on-chip processing area, with magnified views of the regions in the red box shown in **(c)** and **(d)**, demonstrating the chip’s highly symmetrical velocity distribution that supports the actual detection. **e** Velocity field and **f** pressure field in the chip’s detection area, indicating that this region can generate both longitudinal and transverse fluid fields for on-chip monodisperse bead detection. Color legends indicate velocity (µm/s) and pressure (Pa)

The chip’s functional structure is detailed in Fig. 2a, highlighting the three inlets (green circles), the on-chip incubation chambers (red boxes), and the monodisperse detection regions (yellow boxes). To validate the design, COMSOL simulations were performed. The results show a highly symmetrical velocity field distribution in the on-chip processing area (Fig. 2b–d), which supports the reliability of the parallel assays. Furthermore, simulations of the detection region’s velocity (Fig. 2e) and pressure fields (Fig. 2f) confirm that the micropillar array generates both longitudinal and transverse fluid fields, which is essential for achieving the on-chip monodisperse trapping of microbeads required for accurate imaging.

### Characterization of microfluidic chip and isolated EVs

**Fig. 3:**
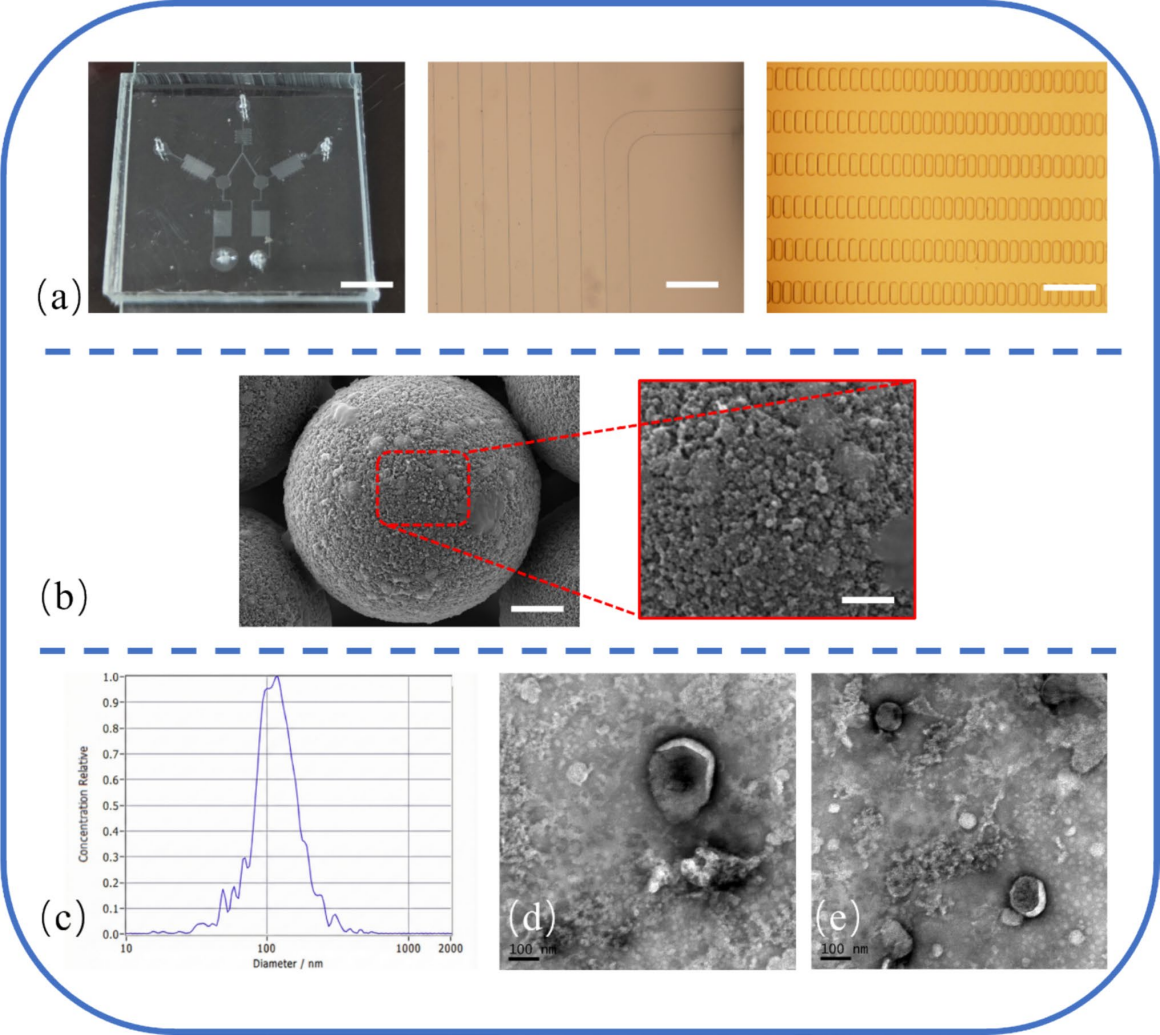
Characterization of the fabricated microfluidic chip and characterization of isolated extracellular vesicles. **a** Photograph of the actual PDMS-glass bonded device with its self-validating structure (left, scale bar: 1 cm), the micrograph of the mixing channels (middle, scale bar: 250 µm) and the detection area, composed by microarrays (right, scale bar: 300 µm), **b** Scanning Electron Microscopy (SEM) image of a 15 µm microbead after on-chip incubation, scale bar is 1 µm. Inset shows captured nanoscale vesicles, scale bar is 500 nm. **c** Nanoparticle Tracking Analysis (NTA) of the isolated SiHa EV population, showing a characteristic peak size distribution around 120 nm (Y-axis label “Concentration Relative”). **d**, **e** Transmission Electron Microscopy (TEM) micrograph of isolated EVs, showing the classic cup-shaped or round-shaped morphology

A photograph of the fabricated microfluidic chip with its self-validating dual-channel structure is shown in Fig. 3a, left and the micrograph of mixing channel and the detection area that composed by microarrays are demonstrated in Fig. 3a, middle and right. To confirm the successful isolation of EVs for assay validation, a comprehensive characterization was performed. Scanning Electron Microscopy (SEM) revealed the successful capture of nanoscale vesicles on the surface of anti-CD63 immuno-magnetic beads (Fig. 3b). Nanoparticle Tracking Analysis (NTA) showed a particle size distribution of SiHa EVs with a distinct unimodal peak around 120 nm, consistent with the expected size range (Fig. 3c). Comparison of NTA profiles across all four cell lines (Fig. S3) confirmed consistent EV size distributions, and justifying the use of normalized particle numbers for quantitative assays. Further, Transmission Electron Microscopy (TEM) confirmed the classic “cup-shaped” morphology characteristic of EVs (Fig. 3d). These results collectively validated the identity of the isolated particles as an EV population.

### System optimization and performance analysis

**Fig. 4:**
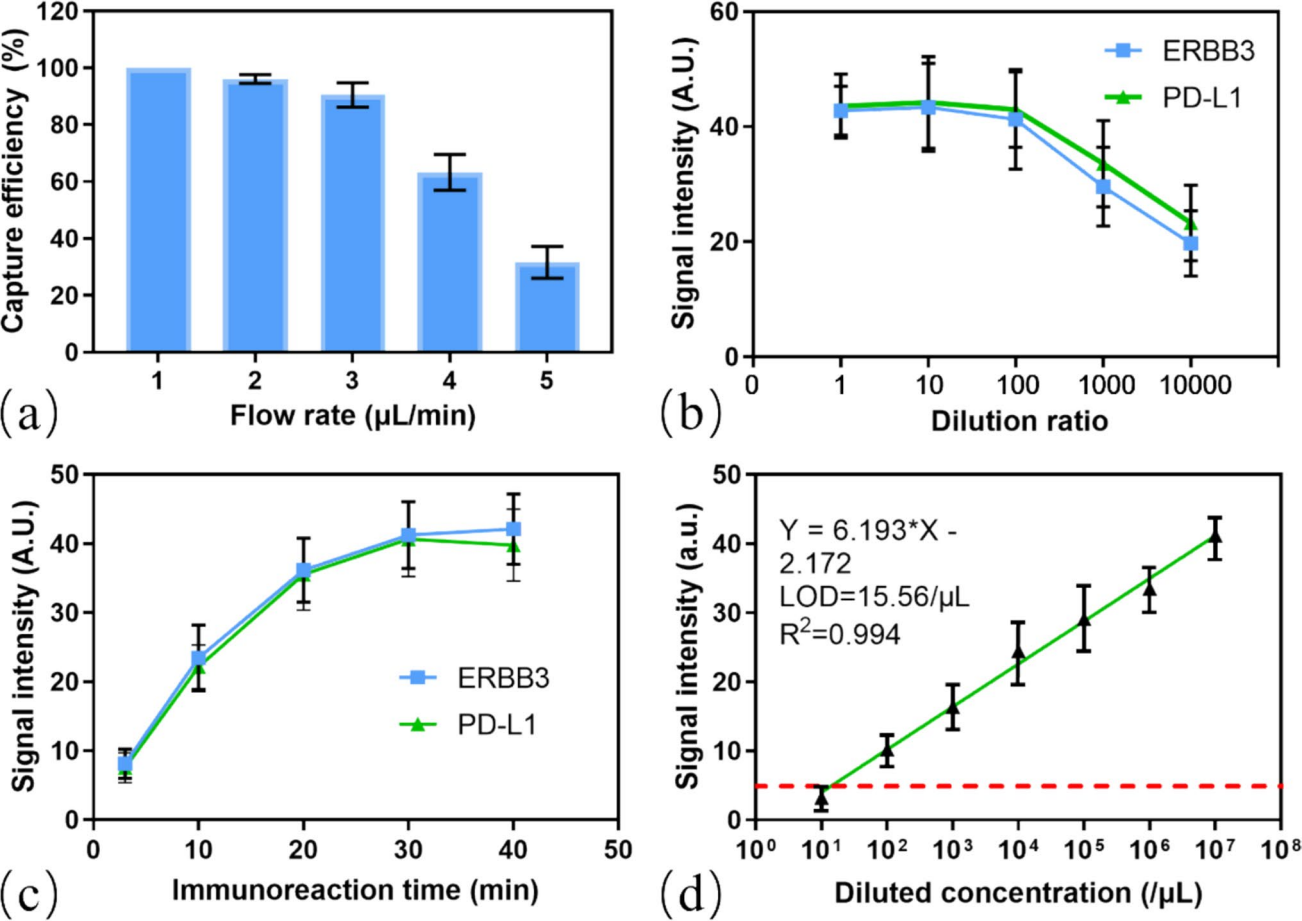
Optimization of the system. **a** Relationship between flow rate and microbead retention efficiency, showing 100% capture at 1 µL/min. The retention efficiency was calculated by counting beads retained in the chamber versus total beads (including those washed out). Error bars represent mean ± SD (n = 3). **b** Signal intensity versus dilution ratio for ERBB3 and PD-L1 probes (X-axis: Probe Dilution Ratio), with the optimal signal achieved at a 1:100 dilution. **c** Relationship between on-chip immunoreaction time and signal intensity, with the signal peaking at 30 min of incubation. **d** Limit of detection for the system determined by serial dilution of the sample for PD-L1 detection (X-axis: EV samples from gradient dilution, concentrations (particles/µL) was measured by NTA), yielding an LOD of 15.56 particles/µL with R^2^ = 0.994. Error bars represent mean ± SD (n = 3)

Key operational parameters were systematically optimized to maximize analytical performance. The efficiency of bead capture and retention in the incubation chamber (microbead retention efficiency) was found to be dependent on the injection flow velocity. As shown in Fig. 4a, retention efficiency remained near 100% at lower flow rates. Although the bead trapping principle is similar to our previous findings [44], the hydraulic resistance of the triple-inlet geometry required independent validation. 1 µL/min was selected as the optimal flow rate to prevent premature washout. The concentration of the detection probes for ERBB3 and PD-L1 was titrated to achieve a maximal signal intensity, with a dilution ratio of 1:100 yielding the best signal intensity for both (Fig. 4b). The on-chip immunoreaction time was also investigated; the fluorescence signal for both markers increased rapidly and reached a plateau within approximately 30 min, which was chosen as the optimal incubation time (Fig. 4c). Under these optimized conditions, the limit of detection (LOD) of the system was determined by analyzing a serial dilution of EVs derived from the SiHa cell line, with their concentration quantified by NTA. As depicted in Fig. 4d, a strong linear correlation (R^2^ = 0.994) was observed between the fluorescence intensity of PD-L1 and the EV concentration. Based on the signal-to-noise ratio at the lowest concentrations, with cell culture medium serving as a blank control, the LOD was calculated to be 15.56 particles/μL. This high sensitivity is attributed to the combination of efficient on-chip processing, the high signal-to-noise ratio of the fluorescent probes, and the precision of the automated quantification algorithm.

To confirm the specificity of the immunoassay, control experiments were performed (Supplementary Materials, Fig. S1). These included incubating anti-CD63-negative beads with EV-rich supernatant and probes, and anti-CD63-positive beads with blank medium and probes. In both cases, negligible fluorescence signals were detected, confirming minimal non-specific adsorption and high assay specificity.

### Automated data quantification by the YOLOv8-based model

**Fig. 5:**
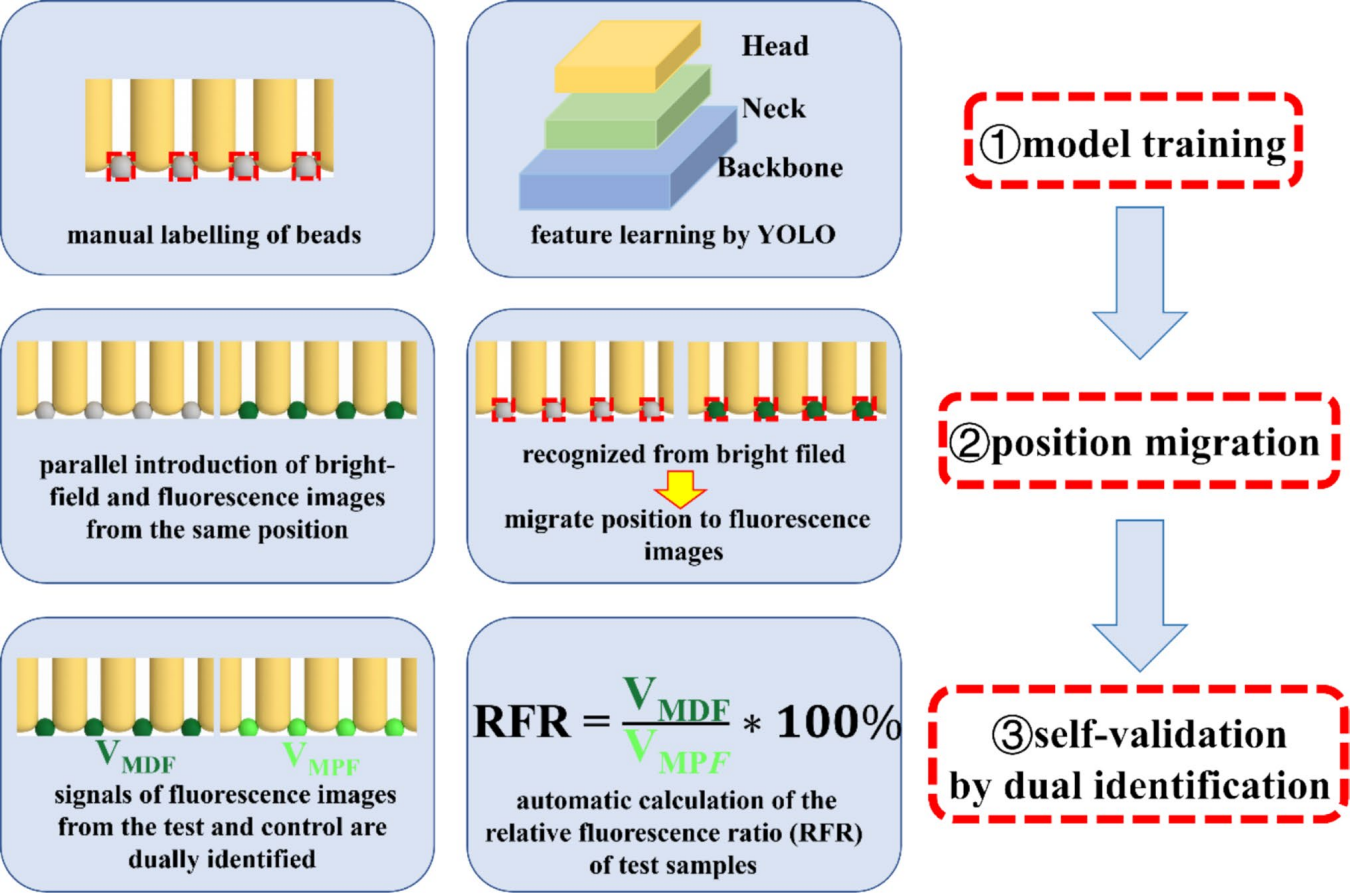
The YOLO-based intelligent image processing workflow. ① Model training, where features are learned from manually annotated microbeads. ② The “position migration” algorithm, which uses parallel bright-field and fluorescence images to identify bead positions in the bright-field and quantify the fluorescence grayscale value within the same circular region in the fluorescence field. ③ On-chip self-validated detection based on dual analysis, simultaneously measuring the test sample and the positive sample to calculate the RFR (Relative Fluorescence Ratio = V_MDF_/V_MRF_ × 100%)

To achieve high-throughput and unbiased analysis, the trained YOLOv8 model was implemented (Fig. 5). For model training, microbead images were manually labelled to teach the model their features. During validation, the model demonstrated excellent performance and signifying its utility in identifying microbeads. The automated workflow begins with the high-confidence (~ 0.8) identification of all microbeads in the bright-field image. The model’s performance was rigorously validated (Supplementary Materials, Fig. S2), achieving an accuracy of ~ 100%. A confusion matrix (Fig. S2a) confirms the model’s high accuracy in distinguishing microbeads from the background.

This exceptional performance is attributed to the highly standardized experimental conditions inherent to our microfluidic platform. Unlike unconstrained object detection tasks in natural scenes, the microbeads possess a strictly uniform geometry (15 µm spheres) and are captured within fixed microfluidic structures under constant illumination. These controlled conditions eliminate common interferences such as target occlusion, scale variation, or complex background noise, allowing the YOLOv8 model to achieve the near-perfect robustness required for precise clinical diagnostics. The subsequent fluorescence quantification is an unbiased calculation of the mean grayscale intensity within the confirmed bead boundary. These positional coordinates are then migrated to the parallel fluorescence image for intensity readout. The software automatically segregates the data based on the “test” and “positive reference” zones of the micropillar array. Finally, it performs statistical inference, calculates the mean intensities for both zones, and generates the final quantitative output, including the normalized relative fluorescence ratio (RFR = V_MDF_/V_MRF_ × 100%). This automated pipeline eliminates subjective, manual data analysis and provides a robust, objective result in seconds.

### Multiplexed profiling of EV markers across gynecological cell lines

**Fig. 6:**
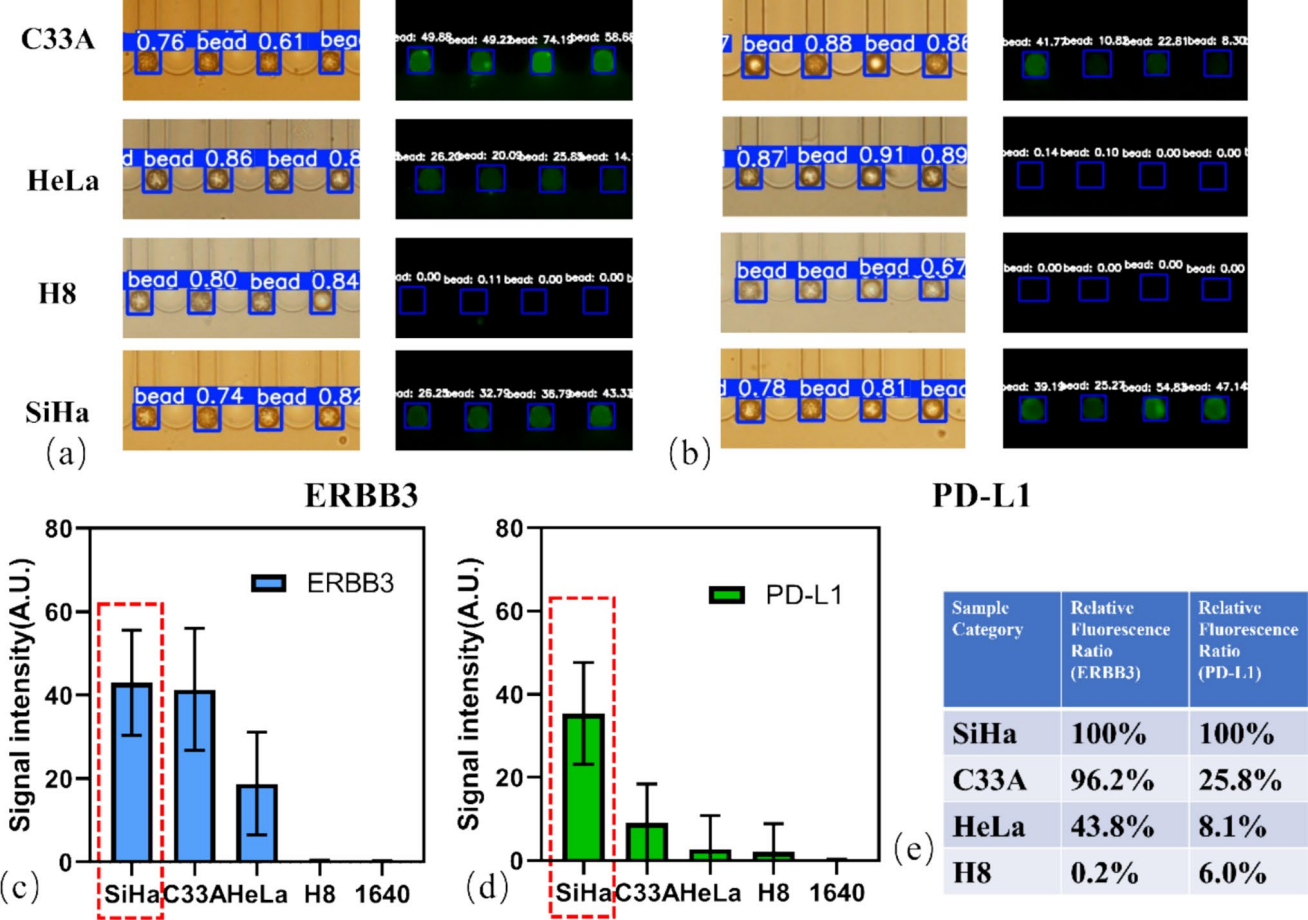
Parallel multiplex detection of ERBB3 **(a)** and PD-L1 **(b)** in C33A, HeLa, H8, and SiHa samples using the integrated microfluidic system. Statistical results are shown in **(c)** and **(d)**, with the SiHa positive reference group indicated by the red dashed box. **e** RFR analysis shows high expression of both ERBB3 and PD-L1 for SiHa, while the other three cell lines exhibit differential abundance ratios. (All experiments performed in triplicate, n = 3, error bars represent mean ± SD)

To demonstrate the platform’s utility in differential diagnostics, a multiplexed assay was performed on EVs from three cervical cancer cell lines (HeLa, SiHa, C33A) and one normal cervical epithelial cell line (H8). Representative fluorescence images for ERBB3 and PD-L1 detection in the four cell lines are shown in Fig. 6a and Fig. 6b, respectively, with the SiHa samples serving as the on-chip positive reference (red dashed boxes). The quantitative data, internally normalized to this reference, revealed distinct and distinguishing protein signatures for each cell line (Fig. 6c, d). (see Supplementary Materials, Fig. S3 for NTA data of all cell lines). Western blot analysis of the EVs (Fig. S3) showed expression trends (SiHa >> HeLa ≈ C33A for PD-L1) that correlated with the on-chip EV RFR results. This biological validation confirms that the microfluidic platform accurately reflects the molecular status of the EVs.

EVs derived from the SiHa cell line (HPV-positive, squamous cell carcinoma), which served as the positive reference, consistently exhibited the highest expression levels of both PD-L1 and ERBB3. In contrast, the HPV-negative C33A cell line showed a markedly different profile, with negligible PD-L1 expression but moderate-to-high levels of ERBB3. Intriguingly, the HeLa cell line (HPV-positive, adenocarcinoma) presented low levels of PD-L1, a result more aligned with the HPV-negative C33A line than the HPV-positive SiHa line, while still maintaining moderate ERBB3 expression. As expected, the non-cancerous H8 epithelial cell line, used as a negative control, showed negligible levels for both markers, confirming the specificity of the assay. The final RFR analysis (Fig. 6e) summarizes these findings. This successful differentiation, particularly the ability to distinguish between the two HPV-positive cell lines, underscores the system’s capacity to generate specific proteomic fingerprints from complex biological samples, highlighting its potential utility in classifying tumor subtypes with high resolution.

### Discussion

Our self-validated microfluidic platform successfully delineated distinct ERBB3/PD-L1 EV signatures among different cervical cancer cell lines, and the biological rationale for these findings is strong. The ability of our platform to quantify these nuanced differences is significant. ERBB3, a known oncogenic driver, was expressed at higher levels in all three cancer cell lines compared to the non-cancerous H8 line, demonstrating the chip’s sensitive and effective detection of a general cancer-associated marker.

The differential expression of PD-L1, a crucial immune checkpoint protein, reveals a more complex and clinically relevant picture. The high EV-associated PD-L1 levels observed in the HPV-positive SiHa line are mechanistically sound, this finding is consistent with the established role of HPV E6/E7 oncoproteins in upregulating PD-L1 expression, thereby creating an immunosuppressive microenvironment. Conversely, the low EV-associated PD-L1 in the HPV-negative C33A line is also biologically informative, suggesting its tumorigenesis may not rely as heavily on the PD-1/PD-L1 axis for immune evasion. Moreover, our system detected low levels of EV-associated PD-L1 from HeLa cells, which are also HPV-positive. This seemingly contradictory result, which is consistent with other report [45], highlights a crucial aspect of tumor biology: the regulation of PD-L1 is multifactorial and may not solely dependent on HPV status. While HPV oncoproteins can induce PD-L1, other cell-intrinsic pathways, genetic and epigenetic modifications, and the influence of the tumor microenvironment can override or modulate this effect. The unique genetic landscape of the HeLa cell line, which has been cultured for decades, may harbor specific regulatory mechanisms that lead to a down-regulation of PD-L1 expression. Furthermore, our on-chip EV measurements are corroborated by Western blot analysis of the EVs (Supplementary Materials, Fig. S3), which similarly shows high PD-L1 expression in SiHa cells and lower levels in C33A and HeLa, strengthening the validity of our platform’s findings. This finding demonstrates that genotype (i.e., HPV status) does not always predict phenotype (i.e., protein expression). Therefore, direct quantitative measurement of biomarkers is indispensable.

While this study utilized cell culture supernatants with relatively consistent properties, we acknowledge that future clinical samples, such as plasma, may exhibit variations in viscosity. Therefore, the proposed platform is well-positioned to mitigate these challenges in translational settings. The system’s robustness is ensured by standard pre-analytical centrifugation and the use of high-precision syringe pumps that maintain constant flow independent of fluid resistance. Furthermore, the microfluidic architecture, which introduces detection reagents via a central inlet into high-surface-area mixing channels, promotes efficient interaction kinetics that minimizes the impact of bulk viscosity. Ultimately, the core self-validated design provides a final layer of correction; by processing a positive reference in parallel, any systemic hydrodynamic fluctuations are normalized in the final Relative Fluorescence Ratio (RFR), ensuring reliable quantification even with complex biological fluids.

It is essential to recognize the inherent trade-off between sensitivity and turnaround time in microfluidic immunoassays. In our previous investigation [15], we successfully employed a sequential injection strategy (capture-wash-label-wash) to minimize steric hindrance, achieving a superior LOD of 10.76 particles/μL. Regrettably, the total processing duration exceeds one hour, restricting its potential for rapid point-of-care applications. Therefore, the current study strategically prioritized speed. Distinct from our previous chip design which focused on the detection of invasion markers (MMP9) [44], the current platform introduces a symmetric triple-inlet architecture. This allows for the simultaneous introduction of a positive reference standard (SiHa supernatant). Furthermore, we upgraded the image analysis algorithm from YOLOv5 to YOLOv8, incorporating a specific module for ratiometric calculation between the parallel test and reference channels. This self-validation mechanism significantly enhances the reliability of the assay by normalizing systematic errors. By adopting a co-incubation protocol, we achieved a acceptable LOD of 15.56 particles/μL within about 45 min, which is a critical optimization for time-sensitive clinical diagnostics. Crucially, to ensure that this co-incubation format does not compromise assay specificity, we validated that the background signal arising from the direct introduction of microbeads and fluorescent probes is negligible (Fig. S1, Control_2). This observation rules out potential false positives caused by the simultaneous presence of reagents, thereby confirming the high specificity of our rapid one-step assay.

Additionally, our self-validated system, which provides an on-chip positive reference for test measurements, ensures that these nuanced and even unexpected biological distinctions are sensitively and stably captured. It effectively normalizes for experimental variations, allowing for the confident differentiation between cell lines and revealing biological heterogeneity which is paramount for developing personalized therapeutic strategies.

## Conclusion

In this work, we have designed and validated a one-stop, integrated, and self-validated microfluidic system for the rapid, sensitive, and automated multiplexed analysis of EV protein markers. Its innovative architecture enables parallel on-chip processing of a test sample against a positive reference, effectively eliminating systematic errors and enhancing analytical reliability. The entire workflow, from sample introduction to result acquisition, is completed within one hour. The platform demonstrated a high degree of sensitivity, achieving a limit of detection of 15.56 particles/μL. Furthermore, the integration of a YOLOv8-based deep learning model ensures accurate, objective, and high-throughput data quantification, removing the potential for human bias.

Our successful application of the system to differentially profile PD-L1 and ERBB3 expression on EVs from distinct cervical cancer cell lines highlights its potential as a powerful tool for cancer diagnostics. Notably, the platform revealed significant heterogeneity in PD-L1 expression even among HPV-positive cell lines, demonstrating its capability to capture subtle but critical phenotypic differences that go beyond simple genetic markers. This underscores the necessity of direct protein measurement for a precise understanding of a tumor’s immune profile. While this study successfully demonstrated the system’s capabilities using cell lines, we acknowledge that its performance has yet to be validated with more complex clinical samples, such as patient plasma or basin eluate. Future work will focus on adapting the workflow for the direct analysis of clinical samples and expanding the panel of detectable biomarkers. In summary, this microfluidic system represents a prospective step towards the realization of rapid, reliable, and high-resolution point-of-care liquid biopsies for cancer and other diseases.

## Supplementary Information

Below is the link to the electronic supplementary material.

Supplementary file1 (DOCX 2006 KB)

## Data Availability

The datasets used and analyzed during this study are available from the corresponding author on request.

## References

[1] C.A. Burmeister, S.F. Khan, G. Schafer, N. Mbatani, T. Adams, J. Moodley, S. Prince, Cervical cancer therapies: current challenges and future perspectives. Tumour Virus Res. **13**, 200238 (2022) 35460940 10.1016/j.tvr.2022.200238 PMC9062473

[2] L. Chang, J. Ni, Y. Zhu, B. Pang, P. Graham, H. Zhang, Y. Li, Liquid biopsy in ovarian cancer: recent advances in circulating extracellular vesicle detection for early diagnosis and monitoring progression. Theranostics **9**(14), 4130–4140 (2019) 31281536 10.7150/thno.34692 PMC6592165

[3] M. Bhadra, M. Sachan, An overview of challenges associated with exosomal miRNA isolation toward liquid biopsy-based ovarian cancer detection. Heliyon **10**(9), e30328 (2024) 38707279 10.1016/j.heliyon.2024.e30328 PMC11068823

[4] D. Chen, B. Cai, Y. Zhu, Y. Ma, X. Yu, J. Xiong, J. Shen, W. Tie, Y. Zhang, F. Guo, Targeting histone demethylases JMJD3 and UTX: selenium as a potential therapeutic agent for cervical cancer. Clin. Epigenetics **16**(1), 51 (2024) 38576048 10.1186/s13148-024-01665-3 PMC10993516

[5] X. He, Q. Dong, C. Weng, J. Gu, Q. Yang, G. Yang, Trends in incidence, survival and initial treatments of gynecological sarcoma: a retrospective analysis of the United States subpopulation. BMC Womens Health **23**(1), 10 (2023) 36624439 10.1186/s12905-023-02161-1 PMC9830743

[6] C. Herrero, A. de la Fuente, C. Casas-Arozamena, V. Sebastian, M. Prieto, M. Arruebo, A. Abalo, E. Colas, G. Moreno-Bueno, A. Gil-Moreno, A. Vilar, J. Cueva, M. Abal, L. Muinelo-Romay, Extracellular vesicles-based biomarkers represent a promising liquid biopsy in endometrial cancer. Cancers (Basel) (2019). 10.3390/cancers11122000 31842290 10.3390/cancers11122000 PMC6966595

[7] Y. Lee, J. Ni, J. Beretov, V.C. Wasinger, P. Graham, Y. Li, Recent advances of small extracellular vesicle biomarkers in breast cancer diagnosis and prognosis. Mol. Cancer **22**(1), 33 (2023) 36797736 10.1186/s12943-023-01741-x PMC9933347

[8] A. Lv, Z. Tu, Y. Huang, W. Lu, B. Xie, Circulating exosomal miR-125a-5p as a novel biomarker for cervical cancer. Oncol. Lett. **21**(1), 54 (2021) 33281965 10.3892/ol.2020.12316 PMC7709555

[9] A. Testa, E. Venturelli, M.F. Brizzi, Extracellular vesicles: new tools for early diagnosis of breast and genitourinary cancers. Int. J. Mol. Sci. (2021). 10.3390/ijms22168430 34445131 10.3390/ijms22168430 PMC8395117

[10] Y. Xue, X. Feng, X. Fan, G. Zhu, J. McLaughlan, W. Zhang, X. Chen, Extracellular vesicles for the diagnosis of cancers. Small Structures (2021). 10.1002/sstr.202100096

[11] H. Al-Madhagi, The landscape of exosomes biogenesis to clinical applications. Int. J. Nanomed. **19**, 3657–3675 (2024) 10.2147/IJN.S463296 PMC11048319 38681093

[12] E. Beit-Yannai, Exosomes in ocular health and disease. Curr. Eye Res. (2025). 10.1080/02713683.2025.2515996 40698627 10.1080/02713683.2025.2515996

[13] N. Dilsiz, A comprehensive review on recent advances in exosome isolation and characterization: toward clinical applications. Transl. Oncol. **50**, 102121 (2024) 39278189 10.1016/j.tranon.2024.102121 PMC11418158

[14] G. Chen, A.C. Huang, W. Zhang, G. Zhang, M. Wu, W. Xu, Z. Yu, J. Yang, B. Wang, H. Sun, H. Xia, Q. Man, W. Zhong, L.F. Antelo, B. Wu, X. Xiong, X. Liu, L. Guan, T. Li, S. Liu, R. Yang, Y. Lu, L. Dong, S. McGettigan, R. Somasundaram, R. Radhakrishnan, G. Mills, Y. Lu, J. Kim, Y.H. Chen, H. Dong, Y. Zhao, G.C. Karakousis, T.C. Mitchell, L.M. Schuchter, M. Herlyn, E.J. Wherry, X. Xu, W. Guo, Exosomal PD-L1 contributes to immunosuppression and is associated with anti-PD-1 response. Nature **560**(7718), 382–386 (2018) 30089911 10.1038/s41586-018-0392-8 PMC6095740

[15] Y. Lu, L. Ye, X. Jian, D. Yang, H. Zhang, Z. Tong, Z. Wu, N. Shi, Y. Han, H. Mao, Integrated microfluidic system for isolating exosome and analyzing protein marker PD-L1. Biosens. Bioelectron. **204**, 113879 (2022) 35180692 10.1016/j.bios.2021.113879

[16] Y. Pang, J. Shi, X. Yang, C. Wang, Z. Sun, R. Xiao, Personalized detection of circling exosomal PD-L1 based on Fe_3_O_4_@TiO_2_ isolation and SERS immunoassay. Biosens. Bioelectron. **148**, 111800 (2020) 31678824 10.1016/j.bios.2019.111800

[17] S.Y. Liu, Y. Liao, H. Hosseinifard, S. Imani, Q.L. Wen, Diagnostic role of extracellular vesicles in cancer: a comprehensive systematic review and meta-analysis. Front. Cell Dev. Biol. **9**, 705791 (2021) 34722499 10.3389/fcell.2021.705791 PMC8555429

[18] J.J.J. Liu, D. Liu, S.K.Y. To, A.S.T. Wong, Exosomes in cancer nanomedicine: biotechnological advancements and innovations. Mol. Cancer (2025). 10.1186/s12943-025-02372-0 40481526 10.1186/s12943-025-02372-0 PMC12144782

[19] N. Mukerjee, A. Bhattacharya, S. Maitra, M. Kaur, S. Ganesan, S. Mishra, A. Ashraf, M. Rizwan, K.K. Kesari, T.A. Tabish, N.D. Thorat, Exosome isolation and characterization for advanced diagnostic and therapeutic applications. Mater. Today Bio **31**, 101613 (2025) 40161926 10.1016/j.mtbio.2025.101613 PMC11950786

[20] J. Shen, Z. Ma, J. Xu, T. Xue, X. Lv, G. Zhu, B. Huang, Exosome isolation and detection: from microfluidic chips to nanoplasmonic biosensor. ACS Appl. Mater. Interfaces **16**, 22776–22793 (2024) 38676635 10.1021/acsami.3c19396

[21] X. Zhao, X. Liu, T. Chen, H. Xie, S. Li, Y. Zhang, H. Zhang, Y. Cao, W. Du, X. Feng, X. Liu, Y. Li, P. Chen, Q. Li, B.-F. Liu, Fully integrated centrifugal microfluidics for rapid exosome isolation, glycan analysis, and point-of-care diagnosis. ACS Nano **19**(9), 8948–8965 (2025) 40014808 10.1021/acsnano.4c16988

[22] S. Mishra, M. Kumarasamy, Microfluidics engineering towards personalized oncology-a review. In Vitro Models **2**(3–4), 69–81 (2023) 39871996 10.1007/s44164-023-00054-z PMC11756504

[23] J.M. Nikoloff, M.A. Saucedo-Espinosa, P.S. Dittrich, Microfluidic platform for profiling of extracellular vesicles from single breast cancer cells. Anal. Chem. **95**(3), 1933–1939 (2023) 36608325 10.1021/acs.analchem.2c04106 PMC9878503

[24] S. Hettiarachchi, L. Ouyang, H. Cha, H.H.W.B. Hansen, H. An, N.-T. Nguyen, J. Zhang, Viscoelastic microfluidics for enhanced separation resolution of submicron particles and extracellular vesicles. Nanoscale **16**(7), 3560–3570 (2024) 38289397 10.1039/d3nr05410a

[25] K.H.W. Ho, H. Lai, R. Zhang, H. Chen, W. Yin, X. Yan, S. Xiao, C.Y.K. Lam, Y. Gu, J. Yan, K. Hu, J. Shi, M. Yang, SERS-based droplet microfluidic platform for sensitive and high-throughput detection of cancer exosomes. ACS Sens. **9**(9), 4860–4869 (2024) 39233482 10.1021/acssensors.4c01357

[26] J. Hu, D. Gao, Recent advances in aptamer-based microfluidic biosensors for the isolation, signal amplification and detection of exosomes. Sensors **25**(3), 848 (2025) 39943486 10.3390/s25030848 PMC11820184

[27] N. Li, C. Cheng, D. Wu, Z. Song, B. Wang, G. Li, F. Yang, Immunofluorescent analysis of exosomes using a microchip filled with transparent antibody-conjugated beads for breast cancer liquid biopsy. Anal. Chim. Acta **1345**, 343743 (2025) 40015783 10.1016/j.aca.2025.343743

[28] B. Mun, H. Jeong, R. Kim, B. Gu, J. Kim, H.Y. Son, H.W. Rho, E.-K. Lim, S. Haam, 3D-nanostructured microfluidic device arranged in a herringbone pattern for the highly effective capture of HER2-positive cancer-derived exosomes in urine. Chem. Eng. J. **482**, 148851 (2024)

[29] L. Pang, C. Tian, Q. Wang, Z. Zhao, B. Pan, Z. Luo, S. Wu, X. Li, J. Fan, An integrating microfluidic system for concentration gradient generation of exosomes and exosome-assisted single-cell-derived tumor-sphere formation. ACS Sens. **10**(2), 678–688 (2025) 39866075 10.1021/acssensors.4c01542

[30] C. Wang, J. Qiu, M. Liu, Y. Wang, Y. Yu, H. Liu, Y. Zhang, L. Han, Microfluidic biochips for single‐cell isolation and single‐cell analysis of multiomics and exosomes. Adv. Sci. (2024). 10.1002/advs.202401263 10.1002/advs.202401263 PMC11267386 38767182

[31] S. Zhand, D.M. Goss, Y.Y. Cheng, M.E. Warkiani, Recent advances in microfluidics for nucleic acid analysis of small extracellular vesicles in cancer. Adv. Healthc. Mater. (2024). 10.1002/adhm.202401295 39707658 10.1002/adhm.202401295

[32] Z. Zhang, Y. Zhou, Separation and aggregation of extracellular vesicles by microfluidics. Biomed. Microdevices (2025). 10.1007/s10544-025-00752-3 40545482 10.1007/s10544-025-00752-3

[33] S.S. Abrar, S.A.M. Isa, S.M. Hairon, M.P. Ismail, M. Kadir, Recent advances in applications of machine learning in cervical cancer research: a focus on prediction models. Obstet. Gynecol. Sci. **68**(4), 247–259 (2025) 40441737 10.5468/ogs.25041 PMC12301556

[34] H. Chen, H. Liu, L. Xing, D. Fan, N. Chen, P. Ma, X. Zhang, Deep learning-driven microfluidic-SERS to characterize the heterogeneity in exosomes for classifying non-small cell lung cancer subtypes. ACS Sens. **10**(4), 2872–2882 (2025) 40167999 10.1021/acssensors.4c03621 PMC12038847

[35] H. Sun, W. Xie, Y. Huang, J. Mo, H. Dong, X. Chen, Z. Zhang, J. Shang, Paper microfluidics with deep learning for portable intelligent nucleic acid amplification tests. Talanta **258**, 124470 (2023) 36958098 10.1016/j.talanta.2023.124470 PMC10027307

[36] Y. Wei, S.M.T. Abbasi, N. Mehmood, L. Li, F. Qu, G. Cheng, D. Hu, Y.P. Ho, W. Yuan, H.P. Ho, Deep‐qGFP: a generalist deep learning assisted pipeline for accurate quantification of green fluorescent protein labeled biological samples in microreactors. Small Methods (2023). 10.1002/smtd.202301293 38010980 10.1002/smtd.202301293

[37] J. Kim, H.Y. Son, S. Lee, H.W. Rho, R. Kim, H. Jeong, C. Park, B. Mun, Y. Moon, E. Jeong, E.-K. Lim, S. Haam, Deep learning-assisted monitoring of trastuzumab efficacy in HER2-overexpressing breast cancer via SERS immunoassays of tumor-derived urinary exosomal biomarkers. Biosens. Bioelectron. **258**, 116347 (2024) 38723332 10.1016/j.bios.2024.116347

[38] Y. Lu, H. Wang, Z. Zeng, J. Hui, J. Ji, H. Mao, Q. Shi, X. Yang, A deep learning-based integrated analytical system for tumor exosome on-chip isolation and automated image identification. Talanta Open **11**, 100398 (2025)

[39] H. Quan, S. Wang, X. Xi, Y. Zhang, Y. Ding, Y. Li, J. Lin, Y. Liu, Deep learning enhanced multiplex detection of viable foodborne pathogens in digital microfluidic chip. Biosens. Bioelectron. **245**, 115837 (2024) 38000308 10.1016/j.bios.2023.115837

[40] T. Diwan, G. Anirudh, J.V. Tembhurne, Object detection using YOLO: challenges, architectural successors, datasets and applications. Multimedia Tools Appl. **82**(6), 9243–9275 (2022) 10.1007/s11042-022-13644-y PMC9358372 35968414

[41] D.D. Dixit, T.P. Graf, K.J. McHugh, P.B. Lillehoj, Artificial intelligence-enabled microfluidic cytometer using gravity-driven slug flow for rapid CD4^+^ T cell quantification in whole blood. Microsyst. Nanoeng. (2025). 10.1038/s41378-025-00881-y 40016189 10.1038/s41378-025-00881-y PMC11868388

[42] H.A. Phan, N.D. Pham, L.Q. Do, T.T. Bui, H.H. Nguyen, T.D. Chu, Machine learning-based bead enumeration in microfluidics droplets enhances the reliability of monitoring bead encapsulation toward single-cell sorting applications. Microfluid. Nanofluid. (2024). 10.1007/s10404-024-02748-6

[43] J. Zhong, Q. Cheng, X. Hu, Z. Liu, YOLO adaptive developments in complex natural environments for tiny object detection. Electronics **13**(13), 2525 (2024)

[44] Y. Lu, W. Zhang, Q. Shi, J. Hui, J. Wang, Y. Song, X. Yang, An integrated microfluidic system for one-stop multiplexed exosomal PD-L1 and MMP9 automated analysis with deep learning model YOLO. Micromachines **16**(11), 1208 (2025) 41302727 10.3390/mi16111208 PMC12654492

[45] G. Yin, T. Qi, J. Wei, T. Wang, Z. Wang, Y. Cui, S. Zong, Fluorescence super-resolution imaging chip for gene silencing exosomes. Sensors (Basel) (2023). 10.3390/s24010173 38203034 10.3390/s24010173 PMC10781284

