# Supplementary material for: An integrated microfluidic system for automatic and self-validated analysis of cervical extracellular vesicle markers PD-L1 and ERBB3
Source: Anal Sci. 2026 Mar 16;42(5):299–312. doi: 10.1007/s44211-026-00871-8 (PMC13095953; doi:10.1007/s44211-026-00871-8)
Supplement: Supplementary file 1 — Supplementary file1 (DOCX 2006 KB) [file 44211_2026_871_MOESM1_ESM.docx]

**Supplementary Information**

**An Integrated Microfluidic System for Automatic and Self-validated Analysis of** **Cervical Exosomal Markers PD-L1 and ERBB3**

Yunxing Lu^a^, Han Qin^b^, Wenjing Zhang^c^, Qiang Shi^a^, Jianan Hui^d,e^, Zhenhua Wu^d^, Yiman Song^d,f^, Xiaoyue Yang^b,g*^

Yunxing Lu and Han Qin have contributed equally to this work.

^a^ School of Science and Technology, Shanghai Open University, Shanghai, 200433, China

^b^ The International Peace Maternity and Child Health Hospital, School of Medicine, Shanghai Jiao Tong University, Shanghai 200030, China

^c^ Department of Obstetrics and Gynecology, The Second Affiliated Hospital of Soochow University, Suzhou 215000, China

^d^ State Key Laboratory of Transducer Technology, Shanghai Institute of Microsystem and Information Technology, Chinese Academy of Sciences, Shanghai, 200050, China

^e^ Shanghai Frontier Innovation Research Institute, Shanghai, China

^f^ School of Stomatology, Dalian Medical University，Dalian 116044, China

^g^ Shanghai Key Laboratory of Embryo Original Diseases, Shanghai 200030, China

* Corresponding author.

E-mail addresses: [yangxiaoyue@sjtu.edu.cn](mailto:yangxiaoyue@sjtu.edu.cn) (X. Yang)

**Figure S1. Specificity validation of the on-chip immunoassay.**

**Figure S2. Comprehensive performance validation of the YOLOv8-based object detection model.**

**Figure S3. Characterization of extracellular vesicles (EVs) size distribution and protein expression.**

**
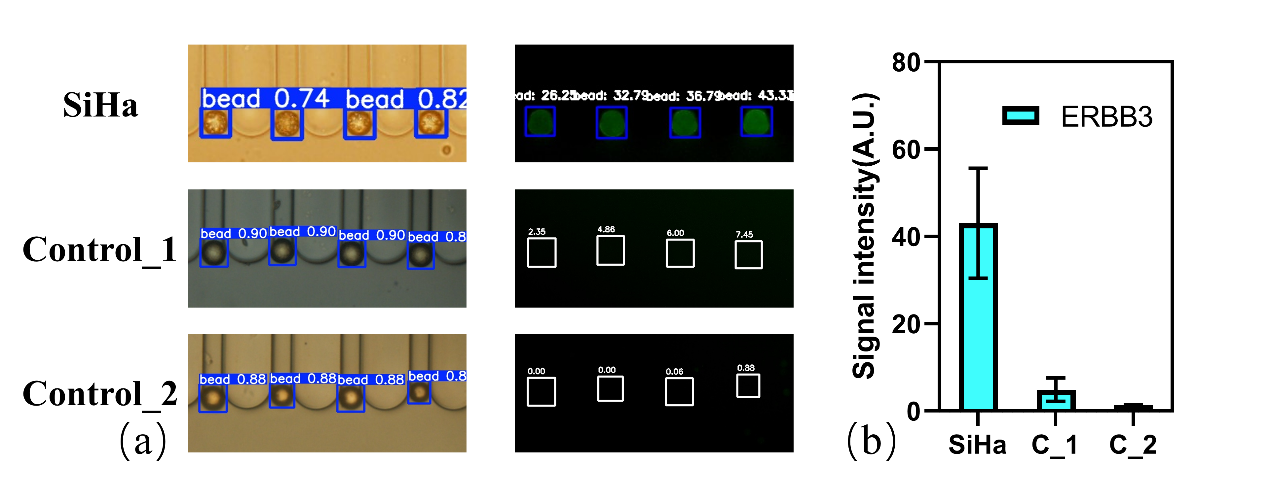
Figure S1. Specificity validation of the on-chip immunoassay.** To exclude false-positive signals caused by non-specific adsorption (NSA), two control experiments were performed: (a) Bead Specificity Control (Control_1): Non-functionalized magnetic beads (without anti-CD63 capture antibodies) were incubated with the SiHa supernatant and fluorescent probes, and Probe Specificity Control (Control_2): Functionalized anti-CD63 beads were incubated with EV-free culture medium and fluorescent probes. (b) Quantified intensities show negligible signals in both control groups compared to the positive assay, confirming that the detected signal is specific to the formation of the Bead-EV-Probe immunocomplex and that NSA of EVs or probes to the channel/beads is minimal. (Data presented as mean ± SD, n=3).

**
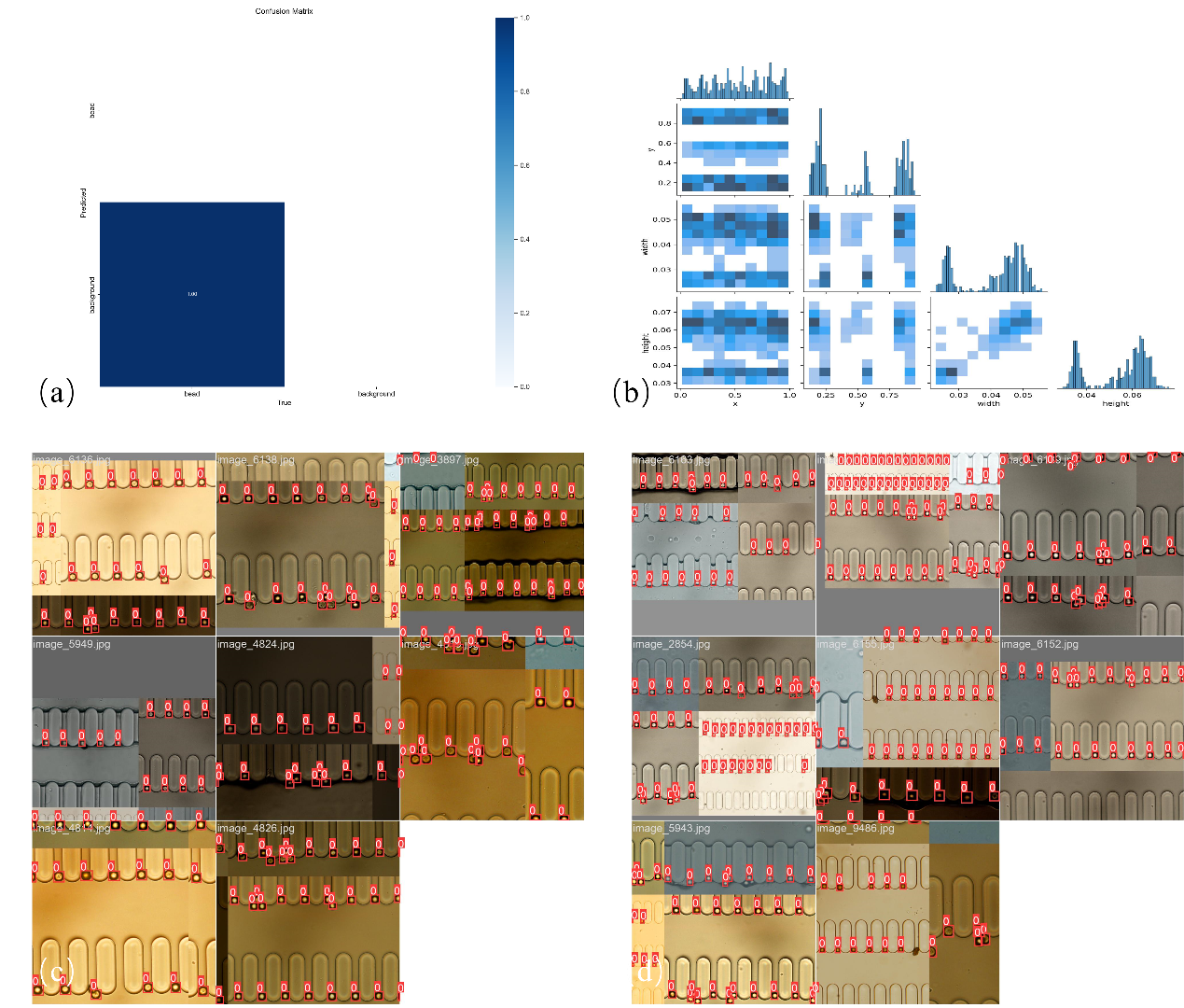
Figure S2. Comprehensive performance validation of the YOLOv8-based object detection model.** **(a)** The normalized confusion matrix evaluated on the dataset, demonstrating high accuracy in distinguishing the target "bead" class from the background. **(b)** Labels correlogram visualizing the spatial distribution (x, y) and dimensional properties (width, height) of the annotated microbeads. The histograms on the diagonal show the frequency distribution, while the scatter plots show the correlation between labels. The tight clustering in the width and height plots reflects the high uniformity of the microbead size. **(c-d)** Representative images from the training batches (Batch 0 and Batch 1), displaying the ground truth bounding boxes overlaid on the bright-field images of the microfluidic channels. These images confirm the model's ability to accurately recognize and localize microbeads across different channel positions and illumination conditions.

**
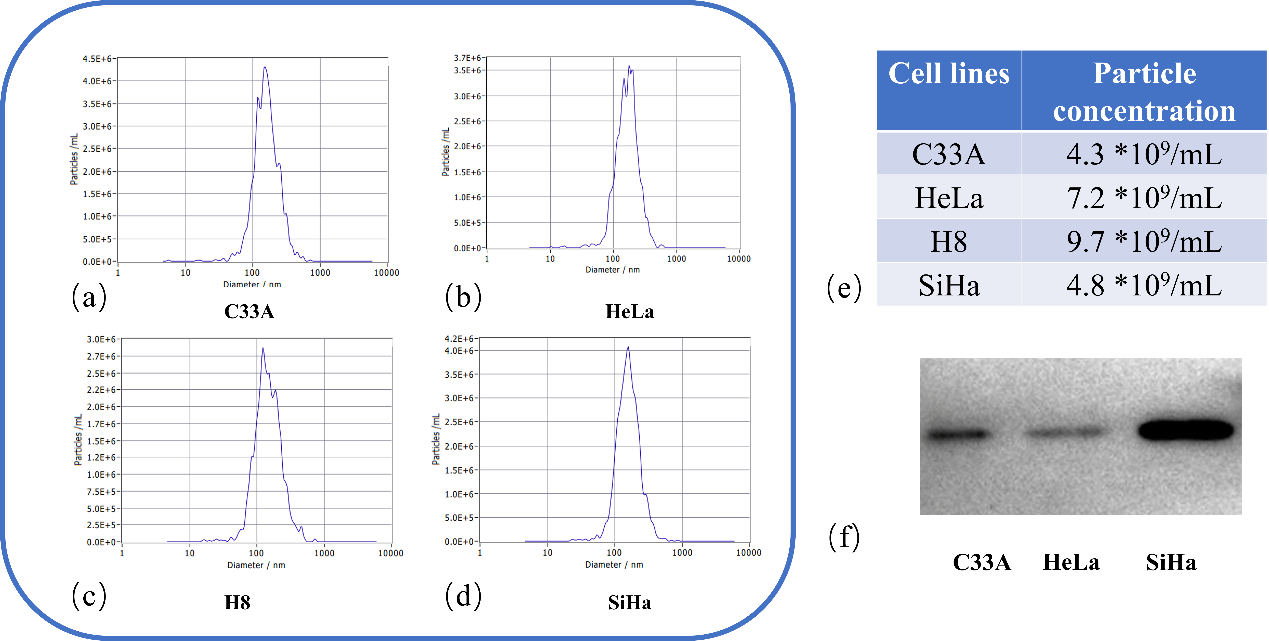
** **Figure S3. Characterization of extracellular vesicles (EVs) size distribution and protein expression.** (a-d) Nanoparticle Tracking Analysis (NTA) size distribution profiles of extracellular vesicles (EVs) isolated from the supernatants of (a) C33A, (b) HeLa, (c) H8, and (d) SiHa cell lines. All samples displayed a typical size distribution with a peak diameter of approximately 120 nm. (e) Summary table listing the classification of the four cell lines and their quantified EV concentrations. To ensure standardized conditions for the microfluidic assay, all EV samples were normalized to a uniform input concentration (1 × 10^9^ particles/mL) based on these NTA measurements. (f) Western blot analysis validating the intrinsic PD-L1 expression levels in the lysates of EVs from three cervical cancer cell lines (C33A, HeLa, and SiHa). The results confirm significantly higher PD-L1 expression in SiHa cells compared to C33A and HeLa cells, providing biological validation for the differential EV profiles observed on the chip.
